# Perylenetetracarboxylic Diimide as Diffusion‐Less Electrode Material for High‐Rate Organic Na‐Ion Batteries

**DOI:** 10.1002/chem.202003624

**Published:** 2020-12-22

**Authors:** Sebastian Liebl, Daniel Werner, Dogukan H. Apaydin, Dominik Wielend, Katharina Geistlinger, Engelbert Portenkirchner

**Affiliations:** ^1^ Institute of Physical Chemistry University of Innsbruck 6020 Innsbruck Austria; ^2^ Institute of Materials Chemistry Vienna University of Technology 1060 Vienna Austria; ^3^ Linz Institute for Organic Solar Cell (LIOS) Institute of Physical Chemistry Johannes Kepler University Linz 4040 Linz Austria; ^4^ Institute for Ion Physics and Applied Physics University of Innsbruck 6020 Innsbruck Austria

**Keywords:** batteries, diffusion-less electrode, organic, perylenetetracarboxylic diimide, sodium

## Abstract

In this work 3,4,9,10‐perylenetetracarboxylic diimide (PTCDI) is investigated as electrode material for organic Na‐ion batteries. Since PTCDI is a widely used industrial pigment, it may turn out to be a cost‐effective, abundant, and environmentally benign cathode material for secondary Na‐ion batteries. Among other carbonyl pigments, PTCDI is especially interesting due to its high Na‐storage capacity in combination with remarkable high rate capabilities. The detailed analysis of cyclic voltammetry measurements reveals a diffusion‐less mechanism, suggesting that Na‐ion storage in the PTCDI film allows for exceptionally fast charging/discharging rates. This finding is further corroborated by galvanostatic sodiation measurements at high rates of 17 C (2.3 A g^−1^), showing that 57 % of the theoretically possible capacity of PTCDI, or 78 mAh g^−1^, are attained in only 3.5 min charging time.

## Graphical abstract

**Fast, faster, dye**: Growing costs and limited resources of lithium has triggered scientific interest in sodium‐ion storage. We demonstrate that a H‐terminated perylenetetracarboxylic diimide (H_2_PTCDI) film allows for remarkably fast charging/discharging rates, governed by a diffusion‐less mechanism. Consequently, exceptionally high sodiation rates are possible, with full sodiation being possible in about 3.5 min is.

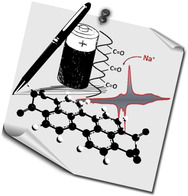

## Introduction

Growing costs and limited resources of Li has triggered substantial scientific interest to find alternatives to the Li‐ion systems. In this respect, the former research on Na‐ion batteries (SIBs) has lately been intensely revived, mostly motivated by the high natural abundance of Na.[^1^, ^2^] Therefore, an important effort is being made in developing high capacity materials for Na‐ion batteries,[^3^, ^4^] which are inherently eco‐efficient and environmentally friendly,^[5]^ in combination with feasible approaches to low‐cost production and recyclability.^[6]^ Organic semiconducting materials, either based on conjugated polymers,^[7]^ or small molecules as the core semiconductor element,^[8]^ hold the promise of delivering low‐cost and energy‐efficient “green” electrodes for Na‐ion batteries.[^9^, ^10^]

Quinones are a fascinating group of these organic battery materials comprising a high theoretical Na‐storage capacity, fast reaction kinetics, and a large structural diversity.^[11]^ These excellent qualities are mainly due to their 1,4‐benzoquinone units. Different types of quinones have been considered for nonaqueous and aqueous organic Na‐ion batteries.[^12^, ^13^, ^14^] These compounds store charge via an ion‐coordination mechanism where the Na ions associate to the negatively charged oxygen atoms upon electrochemical reduction of the carbonyl groups, and dissociate reversibly during the reverse oxidation. Perylene diimides and its derivatives have been previously investigated as cathode material for organic SIBs. In 2014, Luo et al.^[15]^ reported on 3,4,9,10‐perylene‐tetracarboxylicacid‐dianhydride (PTCDA), a commercially available organic pigment, to work efficiently as Na ion battery cathode. In their work a high capacity of 145 mAh g^−1^, high rate capability up to 1000 mA g^−1^, and stable cycling performance over 200 cycles has been reported.^[15]^ In a subsequent work in 2015, also the perylene‐diimide derivative, 3,4,9,10‐perylenetetracarboxylic diimide (PTCDI), which has a hydrogen residual on the nitrogen atom of the imide moiety, has been investigated towards its capability as cathode material in SIBs by Deng et al.^[16]^ Similar to the previously reported PTCDA molecule, the PTCDI molecule is capable of a two‐electron redox reaction with an association/disassociation of two Na ions to the negatively charged oxygen atom of two dicarboximide groups in the potential window of 1–3 V vs. Na/Na^+^. However, during the first cycle of a cyclic voltammetry (CV) measurement only one reduction peak at the potential of 1.6 V was reported. This suggests that a large polarization is needed for the first reduction of the PTCDI molecule. In subsequent cycles three peak couples in the potential range of 3–1.5 V are formed and remain almost constant regarding their potentials and intensities, indicating a reversible, multi‐electron redox reaction. Galvanostatic cycling showed a stable charge/discharge capacity of 138.7/138.6 mAh g^−1^ (constant current of 10 mA g^−1^) after the first cycle, which correlates to a two‐electron redox reaction per PTCDI molecule (giving a theoretical capacity of 137 mAh g^−1^).^[16]^

Organic cathode materials are known for their poor cycle life performance, due to their structural instability particularly at high oxidation potentials and their tendency to dissolve in organic battery electrolytes.^[17]^ In contrast, the long‐term cycling stability of PTCDI is exceptionally enhanced, possibly due to the larger π‐conjugated structure and therefore increased π–π interactions and strong intermolecular H‐bonding of PTCDI molecules.^[8]^ This improves the structural stability of the PTCDI skeletons during repeated sodiation and desodiation cycling.[^18^, ^19^] In addition to its high storage capacity, the PTCDI cathode can also accommodate a remarkable high rate capability, which has only been sparsely investigated and is by now not fully understood. In 1980, Laviron et al.^[20]^ developed a multilayer model for space distributed, redox modified electrodes, which is found to describe the PTCDI–carbon paper (Cp) composite electrode system well. PTCDI on Cp is semiconducting but, due to its morphology and structure, easily accessible for ions to diffuse into.

In this work, by evaluating CV measurements at different scan rates and implementing the theoretical model of Laviron,^[20]^ a mechanistic understanding of the underlying electrochemical process of Na‐ion storage in PTCDI–Cp composite electrodes is gained. Based thereon, a diffusion‐less mechanism can be inferred, suggesting that the diffusion of Na ions into the organic film is extremely fast. It suggests that the transport of the Na counter ion is not the rate‐limiting factor but the electron transfer from the Cp substrate throughout the organic film. Therefore, by utilizing a nanostructured Cp support in combination with a thin PTCDI‐film coverage, very fast sodiation rates can be sustained. As a proof of concept, we show, that at fast sodiation rates of 17 C (2.3 A g^−1^), still 57 % of the theoretical capacity, or 78 mAh g^−1^, of the PTCDI covered Cp electrodes is attained. Full sodiation of a PTCDI–Cp composite electrode in about 3.5 min (17 C) is, to the best of our knowledge, the highest sodiation rate reported for PTCDI by now.

## Results and Discussion

The Cp substrates are coated with a 250 nm thick PTCDI film via a thermal evaporation process. A detailed spectroscopic characterization of the PTCDI film on Cp, by means of Raman spectroscopy, is presented in the Supporting Information (Figure S1).

**Figure 1:**
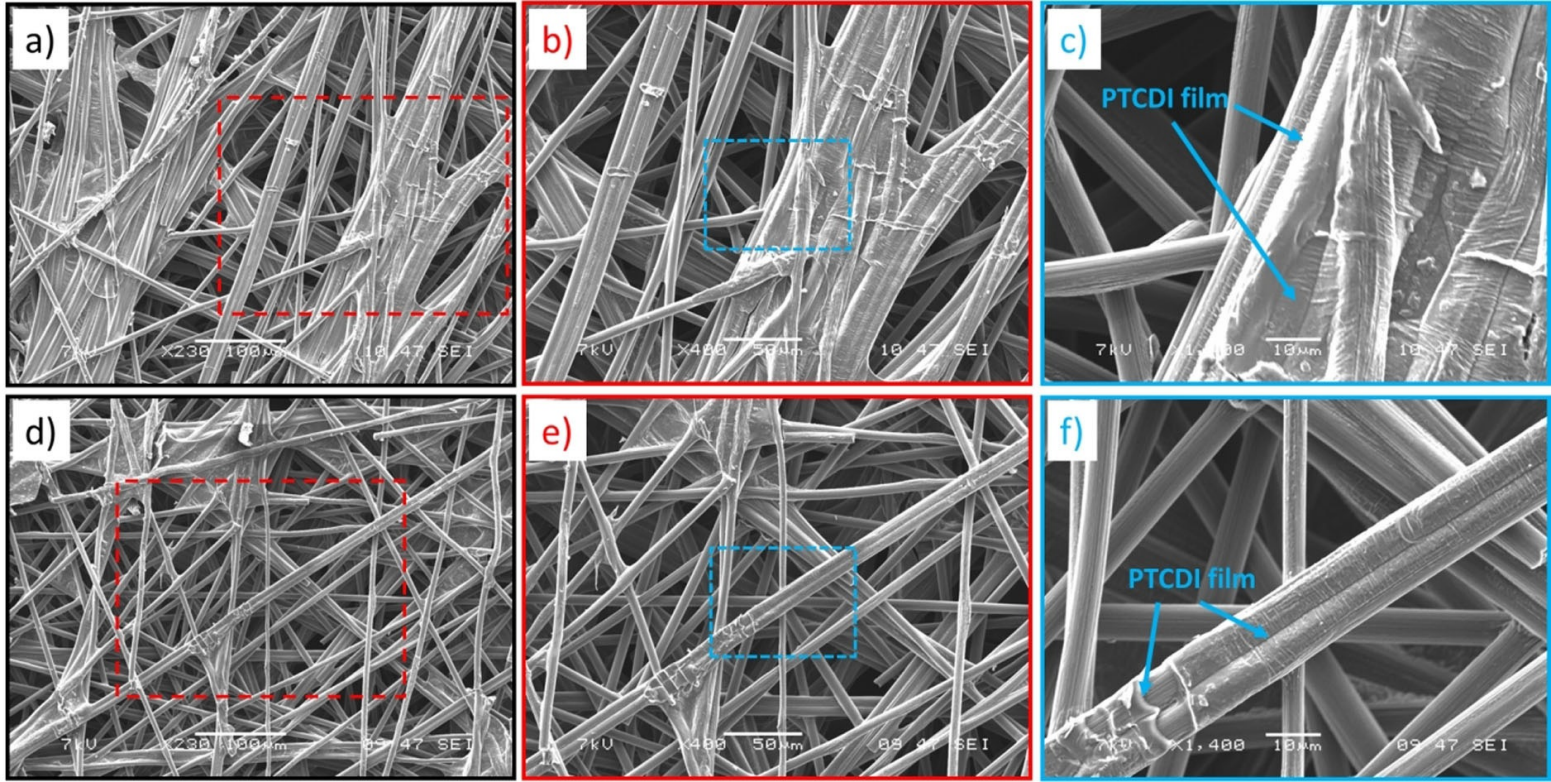
SEM images of PTCDI‐coated (250 nm) carbon paper composite electrodes at different magnifications for two different electrode spots (a–c and d–f).

**Figure 2:**
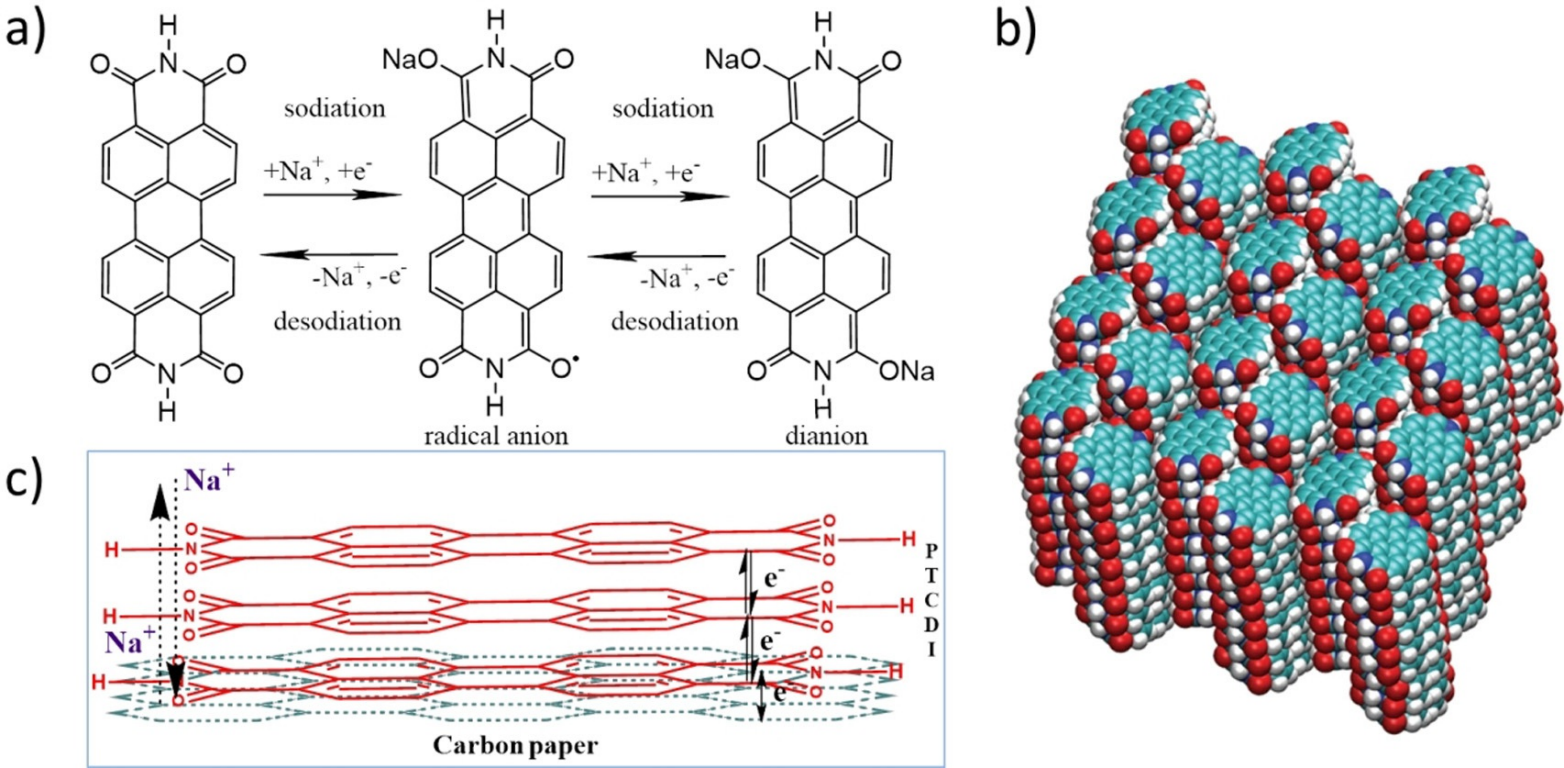
a) Chemical structure of PTCDI and the corresponding two‐electron redox reactions of H_2_PTCDI with the reduction (sodiation) of the neutral molecule to the radical anion and the further reduction (sodiation) to the dianion and both of its back‐oxidations (desodiations) in a sodium electrolyte system. b) Schematic representation of PTCDI bulk crystal structure with white: H, turquoise: C, red: O, and blue: N; reproduced with permission form Ref. [23]. c) Schematic representation of the processes taking place at the working electrode, diffusion of sodium ions, and electron‐transfer reactions.

For a morphological characterization, scanning electron micrograph (SEM) images of PTCDI‐coated (250 nm) Cp electrodes are shown in Figure 1 for two different electrode spots. The low magnification SEM images (Figure 1 a, d) reveal the interconnected network of the carbon fibers forming the supporting Cp substrate. The magnified SEM images in Figure 1 b, e and Figure 1 c, f, focus more on individual carbon fibers. For both electrode spots, the organic film of PTCDI active material covering the subjacent fiber is observed. The thermally evaporated PTCDI film is approximately 250 nm thick and clearly visible as a closed hull, covering the subjacent carbon fiber homogeneously. The organic PTCDI film follows the broad irregularities of the subjacent carbon fiber network. This is in line with previous reports showing that PTCDI and other aromatic ring containing molecules tend to stack one‐dimensionally by π–π interactions to form column structures and 2D stacked layers (Figure 2 b).[^19^, ^21^, ^22^, ^23^]

As reported in Refs. [22] and [24], the H‐terminated PTCDI molecule (H_2_PTCDI) undergoes a reversible two‐electron redox reaction (Figure 2 a) in the potential range from 1 to 3 V measured versus Na/Na^+^. Therefore, two reduction peaks and two back‐oxidation peaks are expected in the CV, corresponding to the reduction of the neutral H_2_PTCDI molecule to the radical anion, the reduction of the radical anion to the dianion, and both the corresponding back‐oxidation reactions (Figure 2 a). The H_2_PTCDI–Cp composite electrode in a Na‐ion battery half‐cell can be thought of as a three‐component system composed of the liquid electrolyte, the solid H_2_PTCDI film, and the solid Cp substrate.

The H_2_PTCDI film, having a thickness of 250 nm, is sandwiched between a liquid/solid interface of the electrolyte and the film and a solid/solid interface of the Cp substrate and the H_2_PTCDI film. As the H_2_PTCDI film is conducting for both, the Na ions and the electrons, the material is considered as a mixed electron‐ion conductor. The liquid/solid interface between the electrolyte and the film is acting as a blocking boundary for the electrons and the solid/solid interface of the film and the carbon paper is acting as blocking boundary for the Na ions (Figure 2 c). For further investigation of the electrochemical characteristics of this system, the battery half‐cell is cycled in a Na containing electrolyte with several different scan rates.

**Figure 3:**
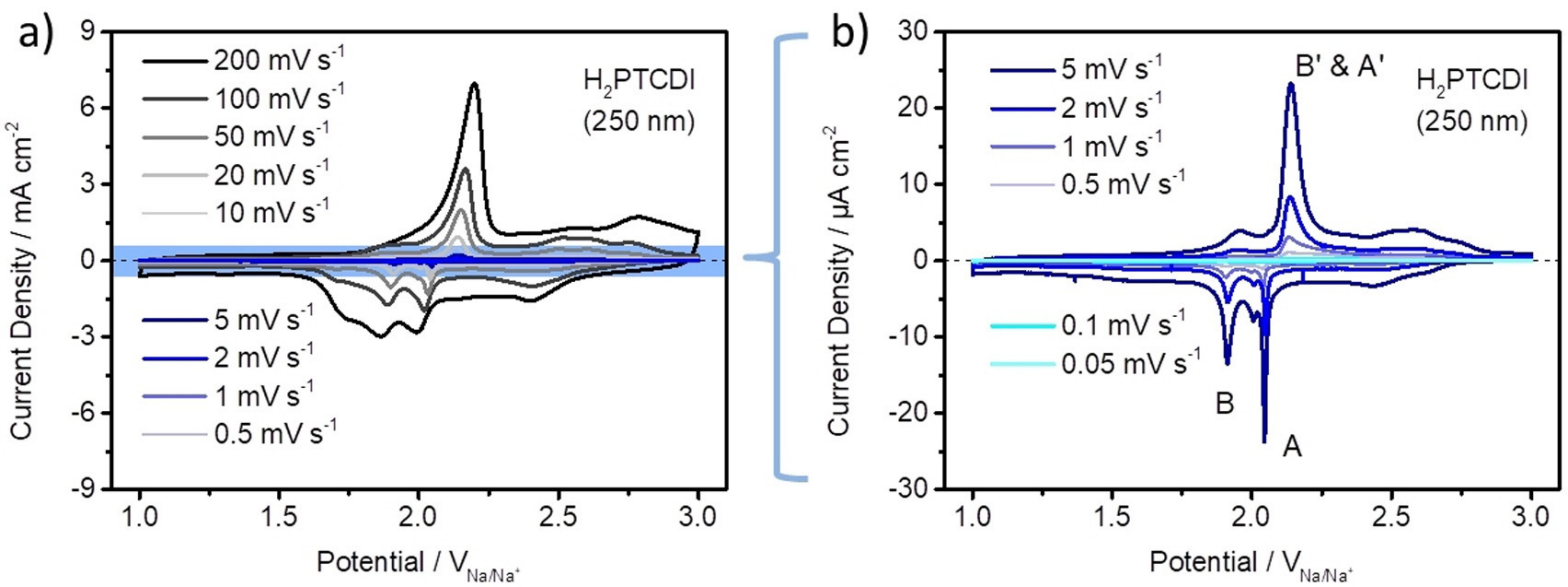
CV measurements of PTCDI (250 nm) on Cp at different scan rates from 200 to 0.05 mV s^−1^ in a 1 m NaFSI/EC/DMC (1:1 (v/v) mixture) electrolyte. For better visibility, the CV measurements at high scan rates from 200 to 10 mV s^−1^ are depicted in (a) and at slow scan rates from 5 to 0.05 mV s^−1^ in (b).

Figure 3 shows CV measurements for different scan rates from 200 to 0.05 mV s^−1^. Two broad reduction peaks and one back‐oxidation peak are visible. Figure 3 b shows a magnification of the CVs with slow scan rates from 5 to 0.05 mV s^−1^. The two reduction peaks denoted as (A) and (B) and the back‐oxidation peaks (B′ & A′) become very sharp, with peak widths (full width at half maximum) of only 15 (A), 45 (B), and 60 mV (B′ & A′) at slow scan rates below 10 mV s^−1^. While in the first cycle only one reduction peak is observable at 1.81 V and one back‐oxidation peak at 2.22 V (Figure S2), in the consecutive cycle the reduction peak splits into two peaks at 1.99 (Figure 3 b, A) and 1.86 V (Figure 3 b, B), corresponding to two one electron reductions of the H_2_PTCDI molecule. The back‐oxidation peak appears at 2.20 V (Figure 3 b, B′ & A′). Comparing the first and the second cycle, it can be seen that the very first reduction of the molecule has a polarization overpotential of about 180 mV (Figure S2) and initially, one reduction reaction comprising two electrons takes place. A possible reason for this polarization overpotential is an initial rearrangement of the H_2_PTCDI molecules to activate the electron transfer and/or an insertion of Na ions into the H_2_PTCDI lattice.^[25]^ The back‐oxidation peak also shows a marginal polarization of 20 mV and the current density of the oxidation peak increases from 5.5 mA cm^−2^ for the first cycle to 6.7 mA cm^−2^ for the second cycle. This suggests that not all H_2_PTCDI molecules are electrochemically active in the first CV cycle. One has to point out that the redox reactions of the H_2_PTCDI molecules in the PTCDI–Cp composite electrode are surprisingly well resolved, even at high scan rates of 200 mV s^−1^, which is not expected when compared to most electrodes tested for Na‐ion batteries.

**Figure 4:**
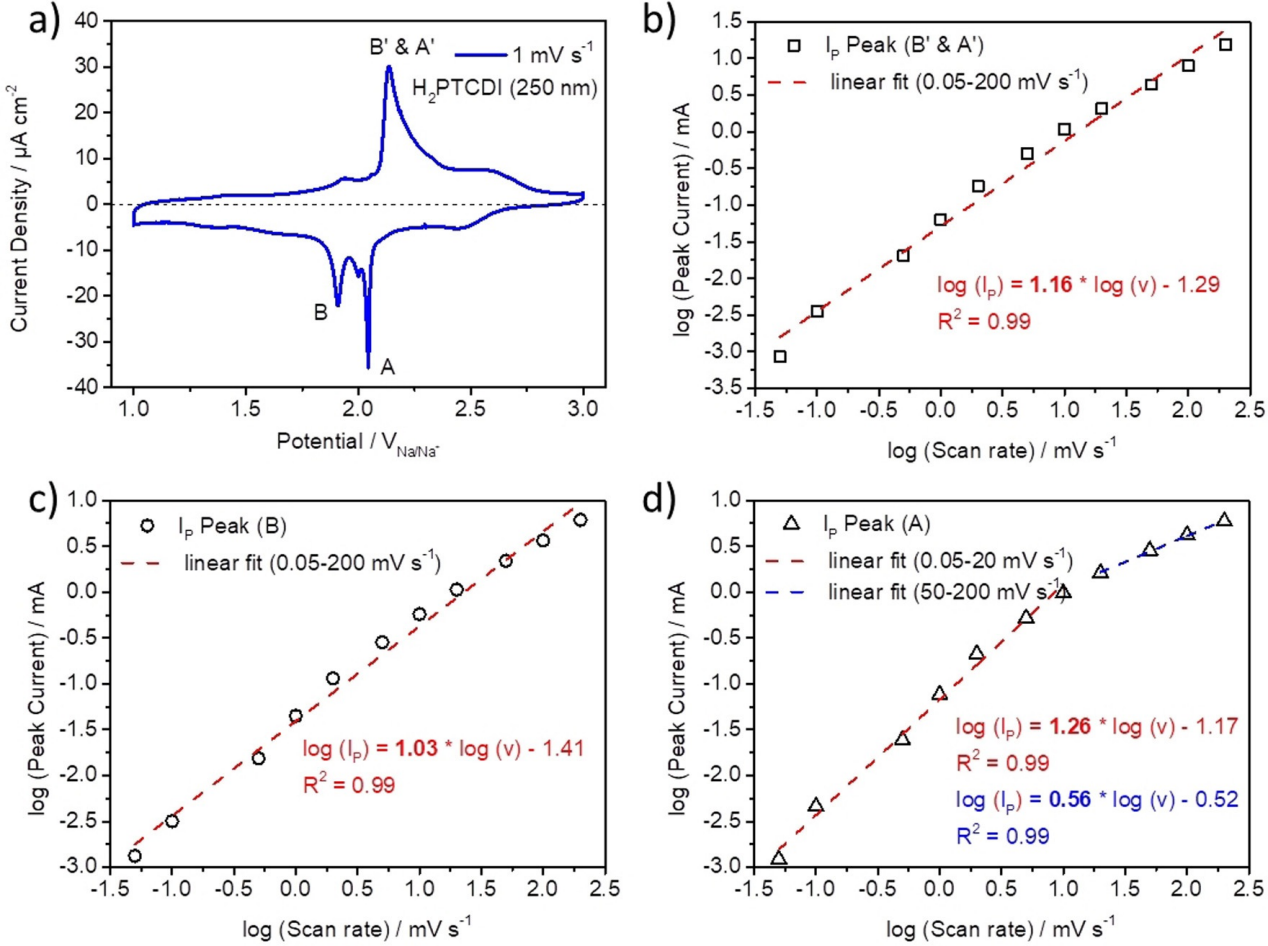
a) Representative CV measurement of the PTCDI–Cp composite electrode at a scan rate of 1 mV s^−1^. Logarithmic plots of the measured peak currents oxidation peak B′ & A′ (b), reduction peak B (c), and reduction peak A (d) against logarithmic applied scan rates. The dashed lines show the linear fits.

For a slow scan rate of 1 mV s^−1^, the first very sharp reduction peak (Figure 3 b, A and Figure 4 a) appears at 2.05 V followed by a second slightly broader reduction peak **(**Figure 3 b, B) at 1.91 V. Interestingly, following the reduction peak (A), a small post‐peak appears (Figure S3). The back‐oxidation peak (Figure 3 b, B′ & A′) at a potential of 2.13 V is tailing. It seems as if the back‐oxidation of the H_2_PTCDI molecule only shows one peak. Either the peak (A′) is a superposition of two peaks (A′ and B′) or, the back‐oxidation is a two‐electron reaction at the potential of 2.13 V. A further, qualitative evolution of the CV curves with decreasing scan rate shows, that the back‐oxidation peak (B′ & A) starts tailing and splits into two separate back‐oxidation peaks (B′) and (A′) with decreasing scan rates. The reduction peaks (A) and (B) also show some tailing for the small scan rates and almost vanish for the slowest scan rate of 0.05 mV s^−1^. The observed behavior, that is, the tailing at small scan rates, may be explained by the kinetic limitation of the electron exchange reaction throughout the film. Laviron et al.^[20]^ mentioned in a theoretical description of a multilayer surface confined electrode, that the kinetic limitation of the electrode exchange reaction throughout the layers is initially observable for slow scan rates, whereas the kinetic limitation of the electrochemical reaction of the first layer with the substrate follows for faster scan rates. Physically this could be interpreted as the H_2_PCTDI molecules in different layers needing a slightly different energy (potential) to be reduced/oxidized, hence the broadening of the peaks. This phenomenon seems to be more significant with decreasing scan rate.

**Figure 5:**
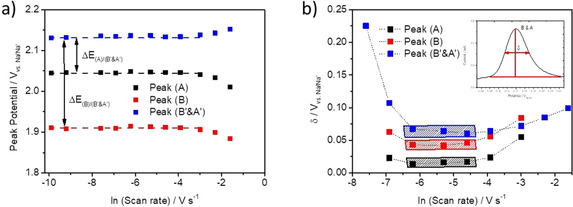
a) Peak potential plotted against logarithmic applied scan rates. Black, red, and blue squares are the values for reduction peak A, reduction peak B, and back‐oxidation peak B′&A′. Dashed lines are guidance for the eyes only. The difference between peak potentials are indicated as Δ*E*_(A)/(B′&A′)_ and Δ*E*_(B)/(B′&A′)_ for the difference between the reduction peak A and the back‐oxidation peak B′&A′ and for the difference between the reduction peak B and back‐oxidation peak B′&A′, respectively. b) Peak half‐height width *δ* plot against logarithmic applied scan rates. Black, red, and blue squares are the δ values for reduction peak A, reduction peak B, and back‐oxidation peak B′ & A′, respectively. The black, red, and blue marked areas are the values used for the evaluation of the interaction energies. Dashed lines are guidance for the eyes only and have no physical meaning. The small insertion illustrates how the *δ* values are obtained.

The CV peaks were further analyzed by plotting the logarithm of the peak current *I*_P_ versus the logarithm of the scan rate (Figure 4 b–d). The values of the peak currents *I*_P_ (Figure 4 b–d) are the absolute values of the respective CV measurements, having the capacitive current for the pure carbon paper substrate without active material (Figure S4) subtracted. Interestingly, the linear fit for the first reduction peak (A) is not uniform over all scan rates but has a slope of about 1 for the scan rates from 0.05–10 mV s^−1^ (Figure 4 d, red dashed line) and a slope of about 0.5 for the faster scan rates from 20–200 mV s^−1^ (Figure 4 d, blue dashed line). The linear fit for the second reduction peak (B) has a slope of about 1 throughout all scan rates (Figure 4 c), and the linear fit of the back‐oxidation peak (B′ & A′) is also characterized by a slope of about 1 for all scan rates (Figure 4 b). A slope of 1 indicates that the current is proportional to the scan rate, representative of a capacitive response,^[26]^ while a slope of 0.5 shows a dependence of the current with the square root of the scan rate. Furthermore the dependence of the peak current with the square root of the scan rate is indicative for a semi‐infinite diffusion behavior,^[27]^ for example, if the diffusion of the Na ions into the H_2_PTCDI film (Figure 2 b) is limiting the peak current. In the H_2_PTCDI system the peak current is mainly proportional to the scan rate. This behavior indicates that the peak current in this system is not limited by the diffusion of the Na ions but rather by either the electrochemical reaction of the first layer of the H_2_PTCDI molecules with the Cp substrate or the electron exchange reaction in the following PTCDI layers (compare Figure 2 c). To further analyze the CV measurements, the peak potentials of the Peaks (A), (B), and (B′&A′) are plotted against the natural logarithm (ln) of the scan rate (Figure 5 a).

The peak potential values are *IR* drop corrected. The resistance (*R*) of the cell was determined by electrochemical impedance spectroscopy (EIS) and is about 3 Ω (compare Figure S5 in the Supporting Information). The peak potentials for the peaks stay constant over a wide range of scan rates (0.05–50 mV s^−1^), with potentials of 2.05, 1.91, and 2.13 V for the peaks (A), (B), and (B′&A′), respectively. For the faster scan rates (100–200 mV s^−1^), a significant deviation from the constant potential is observable, as the reduction peak potentials shift to more cathodic potentials and the oxidation peak potential shifts to a more anodic potential. The constant peak potentials over this wide range of scan rates indicate the reversibility of the redox reaction under these conditions, but unlike the ideal behavior of a reversible surface confined systems the peak potentials of the reduction and oxidation reactions are not the same even for slow scan rates.^[28]^ The difference between the peak potentials, denoted as Δ*E*_(A)/(B′&A′)_ and Δ*E*_(B)/(B′&A′)_, are 88 and 223 mV, respectively. One possible reason for this nonideal behavior may be an attractive intermolecular interaction between the H_2_PTCDI molecules.[^20^, ^28^, ^29^] Neutral, radical anion, and dianion states of the H_2_PTCDI film therefore have different interaction energies. These interaction energies can be investigated by analyzing the half‐height width of the peaks (*δ*) in the CV measurements.[^20^, ^29^]

Figure 5 b shows that *δ* decreases initially with decreasing scan rates, stays nearly constant for the scan rates from 10 to 2 mV s^1^, and increases again for smaller scan rates. The data showing a constant *δ* can be used to calculate the interaction parameters for the H_2_PTCDI film. For all scan rates where no data points are given, the half‐height width was not obtainable from the CVs due to a substantial broadening of the peaks. For a reversible reaction of a surface‐confined electrode, Daifuku et al.^[29]^ gave a theoretical equation that links *δ* with the interaction parameter *Ξ*, Equation 1.

(1) $$\delta =\left(\frac{2RT}{nF}\right){}^{*}\left[\;\ln\left\{\frac{(1+p}{\;(1-p)}\;\right\}-\Xi p\right]$$

with

(2) $$p={\left\{\frac{\left(2-\Xi \right)}{\left(4-\Xi \right)}\right\}\;}^{0.5}$$

and

(3) $$\Xi =\left[\frac{W}{\left(RT\right)}\right]{}^{*}\;\theta_{T}$$

*θ_T_* denotes the surface coverage and is assumed to be 1 for all three different states of the film. *R* is the ideal gas constant with 8.314 J mol^−1^ K^−1^, *T* the temperature taken as 298 K, *n* the number of electrons transferred, and *F* the Faraday constant with 96 485 C mol^−1^. *W* is the interaction energy under investigation, given in kJ mol^−1^. The results for peaks (A), (B), and (B′&A′) are given in Table 1.

**Table 1:** Thermodynamic interaction parameters obtained for a 250 nm‐thick H_2_PCTDI film.

Scan rate	*δ*(A) [V]	Ξ	*W* [kJ mol^−1^]	*δ*(B) [V]	Ξ	*W* [kJ mol^−1^]	*δ*(B‘ & A‘) [V]	Ξ	*W* [kJ mol^−1^]
10	0.017	1.86	4.54	0.0460	0.76	1.85	0.060	1.24	3.01
5	0.016	1.91	4.65	0.0420	0.87	2.12	0.064	1.17	2.85
2	0.014	1.93	4.71	0.0431	0.82	2.00	0.067	1.12	2.72
average			4.63			1.99			2.86
standard deviation			0.08			0.13			0.14

The interaction energies *W* are found to be positive for all three redox states of the H_2_PTCDI‐film—neutral (Table 1, grey), radical‐anion (Table 1, red), and dianion (Table 1, blue). Furthermore, the neutral film has the strongest interactions and the radical‐anion film the weakest, resulting in a sharp peak spike of the first reduction wave of the H_2_PTCDI‐film on Cp (indicated by A in Figure 4 a).^[20]^ The half‐height width increases on increasing scan rates because some irreversibility of the reactions is occurring, as mentioned before. Interestingly, the same seems to be true for small scan rates.

It can be inferred that the presence of Na ions gives rise to a competition between hydrogen bonding in the PTCDI film and the classical ion–dipole interaction, suggesting a lessening in the interaction energies of the neutral molecules. Unfortunately, this lessening in the interaction energies may be adding to the potential material instability upon reduction and the dissolution of active material upon repeated sodiation/desodiation cycling.

**Figure 6:**
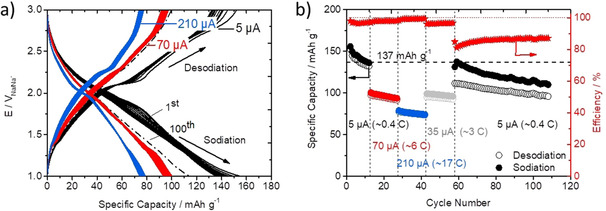
a) Galvanostatic charge/discharge performances of PTCDI (250 nm) on Cp with different applied constant currents of 5 μA (black line), 70 μA (red line), and 210 μA (blue line). b) Specific capacity (charge, open circles; discharge, closed circles) of PTCDI on Cp and corresponding calculated efficiencies versus cycle number at different currents.

Thus, to evaluate the long‐term active capacity of the H_2_PTCDI composite electrodes, galvanostatic cycling with potential limitation (GCPL) measurements were done. Figure 6 shows the GCPL data of a 250 nm thick H_2_PTCDI composite electrode in the potential range from 1 to 3 V, with different applied constant currents of 5 (≈0.4 C, black line), 70 (≈6 C, red line), and 210 μA (≈17 C, blue line).

The experimental response is characterized by a sloping plateau‐like region around the peak potentials A, B, and A′ & B′ that are known from the CV measurements (Figure 3). The most pronounced plateau was measured at the oxidation peak potential A′ & B′ (Figure 6 a). The first cycle has a sodiation capacity of 14.1 μAh and a desodiation capacity of 13.8 μAh, which corresponds to a specific gravimetric capacity of 156 and 152 mAh g^−1^. This is higher than the theoretical capacity of 137 mAh g^−1^ (Figure 6 b, black dashed line) expected for a two‐electron reduction/back‐oxidation of the H_2_PTCDI molecule. The additional capacity can be attributed to the contribution of the Cp paper substrate, which itself has a capacity contribution of 2.6 μAh for an applied constant current of 5 μA (compare Supporting Information, Figure S6 a, dashed black line). The 12^th^ cycle shows a distinct decrease of the plateau‐like regions, which can be attributed to the loss of active material, probably due to dissolution. This is further corroborated by UV/Vis measurements of the battery electrolyte solution after 115 GCPL measurements shown in Figure 6, revealing an absorption maximum at around 570 nm and consequently a distinct color change of the battery electrolyte towards violet (Figure S7). The capacity values for the 12^th^ cycle are 12.4/12.0 μAh or 136/133 mAh g^−1^ for the sodiation/desodiation, respectively (Figure 6 b and in more detail in Figure S6 in the Supporting Information). This corresponds to an active material loss of about 12 % in the first 12 cycles, under the assumption that the other contributions to the capacity remain constant. The 100^th^ cycle (depicted as dashed black line in Figure 6 a) has a specific capacity of 115 mAh g^−1^, which corresponds to a loss of 26 % of active material. In Figure 6 b the obtained values of the specific gravimetric capacity and the corresponding coulombic efficiency over 115 cycles with different applied constant currents of 5 (≈0.4 C), 70 (≈6 C), 210 (≈17 C) and 35 μA (≈3 C) are depicted. The coulombic efficiency for the evolution of the specific gravimetric capacity for the first 12 cycles stays nearly constant with a value of 97 %. For the next 15 cycles a constant current of 70 μA (≈6 C) is applied. The specific capacity in the first cycle at 70 μA is measured with 100/97 mAh g^−1^ for sodiation/desodiation, respectively. After 15 cycles at 70 μA, the composite electrode loses about 7 % of its capacity resulting in a coulombic efficiency of 98 %. When the applied constant current is further raised to 210 μA (≈17 C), the measured specific capacity drops to 78/77 mAh g^−1^ for the first cycle and shows a loss in capacity of 5 % after 15 cycles. The coulombic efficiency is thereby maintained above 99 %. Following, a constant current of 35 μA (≈3 C) was applied for the next 15 cycles. The obtained specific capacity values for its first cycle are 99/95 mAh g^−1^, and a loss of 3 % after 15 cycles at 35 μA is recorded with a coulombic efficiency of 96 %. When the applied current is lowered again to the initial current of 5 μA (≈0.4 C), the sodiation/desodiation capacity is measured with a specific capacity of 138/112 mAh g^−1^, respectively. This capacity is very close to the capacity after the initial 12 cycles at 5 μA, revealing the good cyclability of the H_2_PTCDI–Cp composite electrodes at elevated sodiation rates. The capacity, however, drops significantly to 110/96 mAh g^−1^ after an additional 50 cycles at the applied low constant current of 5 μA, which is a loss of 20 % for the sodiation capacity and of 14 % for the desodiation capacity. This impinges clearly on the coulombic efficiency, which shows a value of only 81 % at its first cycle with 5 μA and of 87 % at its 50^th^ cycle. This severe drop in efficiency is rooted in an irreversible reduction reaction near 1 V in the sodiation cycle, which is best seen in a differential capacity plot for the GCPL data (Figure S8). This irreversible side reaction is most dominant for small currents, for two main reasons. First, its overpotential increases with increasing C rate (beyond 1 V) and secondly, the reduction reaction is most likely linked to an electrolyte decomposition reaction as it can be also seen on the pure Cp substrate. This electrolyte decomposition is found to increase the lower the potential is (the more reductively biased) and the longer the electrode is kept at this low potential. A discharge rate of 0.4 C corresponds to a discharge time of about 2.5 h, while a discharge rate of 6 C corresponds to a discharge time of about 0.17 h or 10 min. The higher the rate, the less time the electrode is kept at low potentials and the less the electrode is suffering from side reactions and material dissolution, leading to higher coulombic efficiencies (Figure 6 b). The capacity resulting from the active material gets lower during cycling while the capacity of the irreversible side reaction does not drop correspondingly; hence, this results in a drop in efficiency with prolonged cycling. Overall, the loss of active material capacity during the whole experiment is 37 % (loss of desodiation capacity).

**Figure 7:**
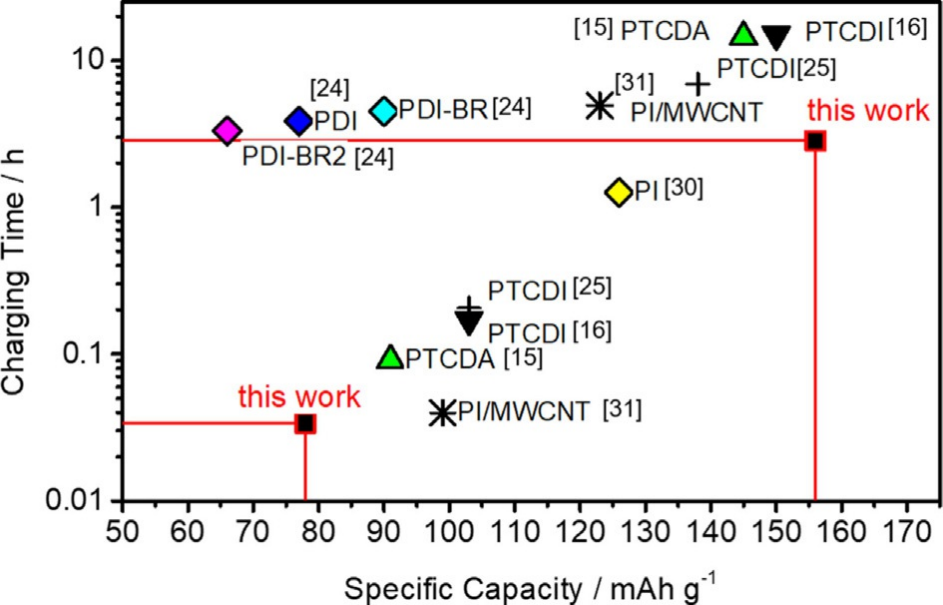
Comparison of the charging time over the specific capacity reached for various perylene‐based cathode materials for Na‐ion batteries. Definitions of the shown materials are: 3,4,9,10‐perylene‐tetracarboxylicacid‐dianhydride (PTCDA), 3,4,9,10‐perylene‐bis(dicarboximide) (PTCDI), N,N′‐bis(n‐propylacetyl)‐perylene‐3,4,9,10‐tetracarboxylic diimide (PDI), *N*,*N*′‐diamino‐3,4,9,10‐perylenetetracarboxylic polyimide (PI) and multiwalled carbon nanotube (MWCNT). The data has been reproduced from several literature references.[^15^, ^16^, ^24^, ^25^, ^30^, ^31^]

The observed cycle‐life performances are relatively good compared to low‐molecular‐weight organic active materials previously reported for lithium or sodium systems, which typically suffer from poor cycle performance due to the dissolution of the redox‐active molecules into the electrolyte solutions.[^14^, ^32^] While there are numerous publications on organic cathode materials for Li‐ and Na‐ion batteries, there is still a manageable amount of literature on perylene‐based cathode materials for Na‐ion batteries. To put the results obtained for our PTCDI‐Cp composite electrodes in perspective, Figure 7 shows a comparison of the charging time over the specific capacity reached for various perylene‐based cathode materials that have been reported for their application in Na‐ion batteries. Among them are 3,4,9,10‐perylene‐tetracarboxylic acid dianhydride (PTCDA),^[15]^ PTCDI for Na‐^[16]^ and Li^[25]^‐based electrolytes, *N*,*N′*‐bis(*n*‐propylacetyl)‐perylene‐3,4,9,10‐tetracarboxylic diimide (PDI),^[24]^ *N*,*N*′‐diamino‐3,4,9,10‐perylenetetracarboxylic poly‐imide (PI),^[30]^ and polyimide (PI)/multi‐walled carbon nanotube (MWCNT) composite electrode.^[31]^ In comparison, it can be seen that our PTCDI‐Cp composite electrodes allow for both, high specific capacity and exceptionally fast charging/discharging rates (Figure 7).

## Conclusions

We demonstrate that Na‐ion storage in the H‐terminated perylenetetracarboxylic diimide (H_2_PTCDI) film allows for remarkably fast charging/discharging rates, governed by a diffusion‐less mechanism, where the transport of the Na counter ion is not a limiting factor. Consequently, exceptionally high sodiation rates of 17 C (2.3 A g^−1^) are possible. Since the PTCDI film is best described by a multilayer surface‐confined electrode, the kinetic limitation of the electrode seems to be governed by the electron exchange reaction throughout the stacked PTCDI layers. All three redox states of the H_2_PTCDI molecules (neutral, radical‐anion, and dianion) are found to have positive interaction energies, explaining the non‐ideal behavior, that is, the hysteresis effect and the observed sharp peaks upon cyclic voltammetry (CV) measurements, compared to a reversible surface confined system. While H_2_PTCDI on carbon paper (Cp) demonstrates reasonably good cycle‐life performances, its dissolution in the battery electrolyte is still a serious issue that has to be addressed in future studies. Similar to inorganic, carbon‐based materials, such as reduced graphene oxide or expanded carbon,[^33^, ^34^] quinone‐based organic composites like H_2_PTCDI on Cp may offer a cost‐effective, abundant, and also environmentally benign cathode material for fast rechargeable Na ion batteries.

## Conflict of interest

The authors declare no conflict of interest.

## Supporting information

As a service to our authors and readers, this journal provides supporting information supplied by the authors. Such materials are peer reviewed and may be re‐organized for online delivery, but are not copy‐edited or typeset. Technical support issues arising from supporting information (other than missing files) should be addressed to the authors.

Supplementary
