# Supplementary material for: Perylenetetracarboxylic Diimide as Diffusion‐Less Electrode Material for High‐Rate Organic Na‐Ion Batteries
Source: Chemistry. 2020 Dec 22;26(72):17559–66. doi: 10.1002/chem.202003624 (PMC7839514; doi:10.1002/chem.202003624)
Supplement: Supplementary file 1 — Supplementary [file CHEM-26-17559-s001.pdf]

Chemistry–A European Journal

Supporting Information

Perylenetetracarboxylic Diimide as Diffusion-Less Electrode Material for High-Rate Organic Na-Ion Batteries

Sebastian Liebl,[a] Daniel Werner,[a] Dogukan H. Apaydin,[b, c] Dominik Wielend,[c] Katharina Geistlinger,[d] and Engelbert Portenkirchner*[a]

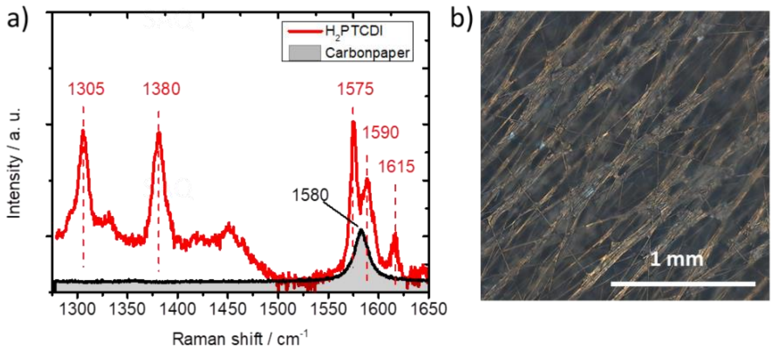

c)

Raman Shift/ cm^{-1}	Assignment
1305	δC–H; νC–C; (δNH)
1380	δC–H; νC–C; (δCH_3)
1575	δC–H; νC–C
1580	νC–C (Graphen)
1590	δC–C; δC–H
1615	νC=O

Figure S1: a) Raman spectra of H_2PTCDI coated (250 nm, red line) carbon paper composite electrodes in comparison to the pure carbon paper substrate (black line). b) optical microscope image (2x2 mm) of the electrode are investigated by Raman spectroscopy in a) and c) peak assignment for the measured Raman signal shown in a).

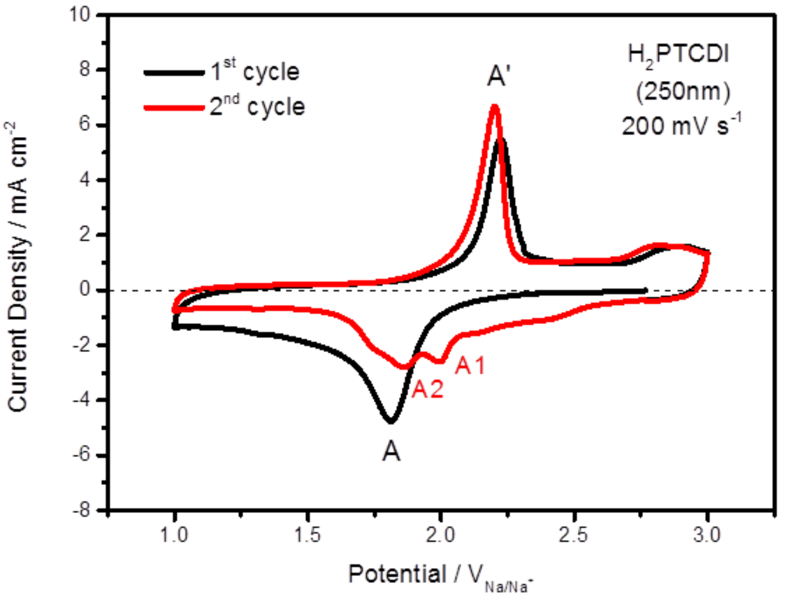

Figure S2: CV of the first two cycles of a 250 nm thick H_2PTCDI composite electrode with a scan rate of 200 mV s^{-1}. First cycle in black and its reduction and oxidation peak is denoted with A and A', respectively. Second cycle is depicted in red and its reduction/back-oxidation peaks are denoted as A1, A2 and A'.

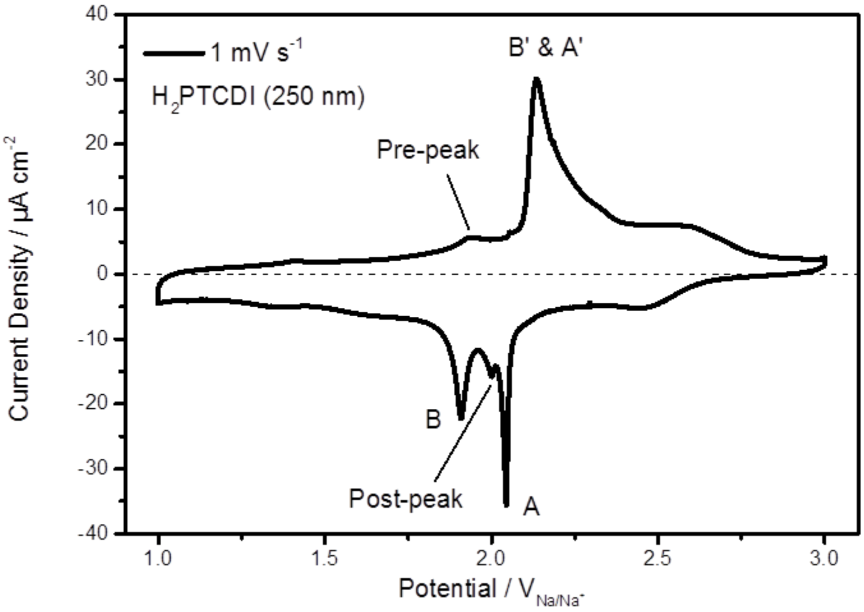

Figure S3: CV of a 250 nm thick H_2PTCDI composite electrode with a scan rate of 1 mV s^{-1}. The two reduction peaks are denoted as A and B. The back-oxidation peak is denoted as B' & A'. A post-peak and a pre-peak are indicated separately.

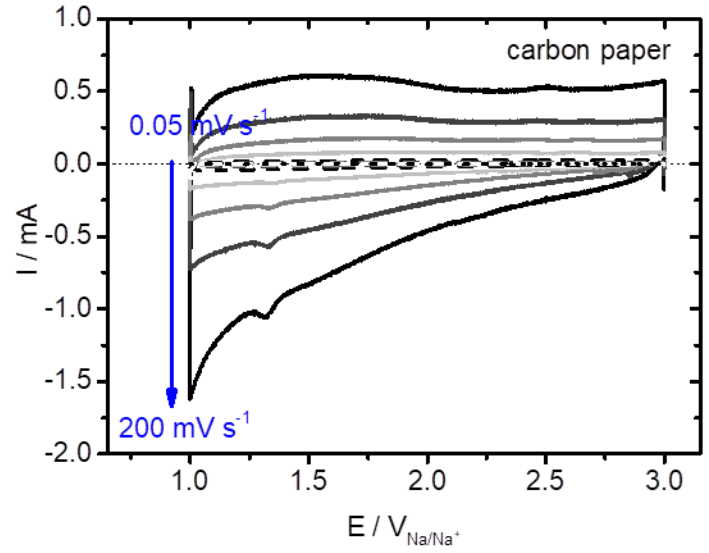

Figure S4: CV measurements of the pure carbon paper (Cp) substrate at different scan rates from 200 to 0.05 mV s^{-1} in a 1 M NaFSI/EC:DMC electrolyte.

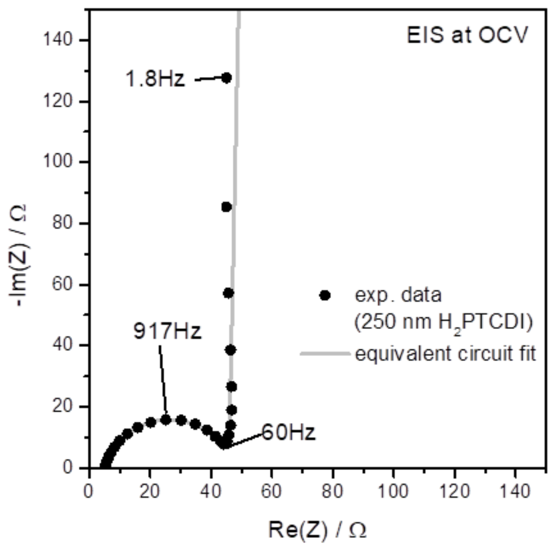

Figure S5: Impedance data plotted as Nyquist plot measured at OCV after assembling the battery half-cell (black data points are for a pristine 250 nm thick H_2PTCDI composite electrode) for IR-drop determination. Frequencies for specific/certain data points are depicted.

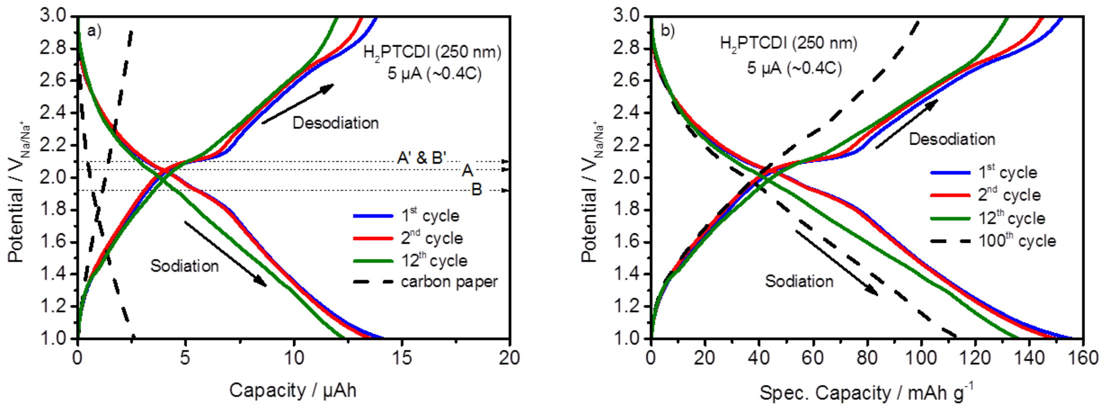

Figure S6: GCPL measurements for H_2PTCDI composite electrodes in the potential range from 1-3 V: a) For a 250 nm thick H_2PTCDI composite electrode with an applied constant current of 5 µA (~0.4C). The first, second and 12th cycle are illustrated. Additionally, the response of the pure carbon paper substrate is shown as dashed black line. The peak potentials A, B and A' & B', obtained from CV measurements, are illustrated as horizontal dashed lines. b) Data of a) converted to the specific capacity in (mAh g^{-1}). Additionally, the 100th cycle is illustrated as dashed line.

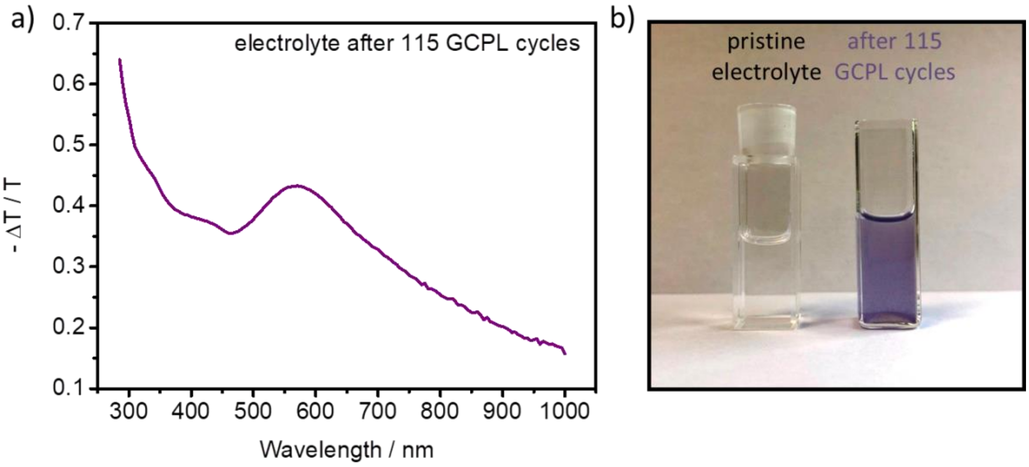

Figure S7. a) UV–vis measurements of the battery electrolyte solution after the 115 GCPL measurements shown in **Figure 6** in the main text from 300 nm to 1000 nm, revealing an absorption maximum at around 570 nm and consequently a distinct color change of the battery electrolyte from the transparent pristine towards deep violet (b).

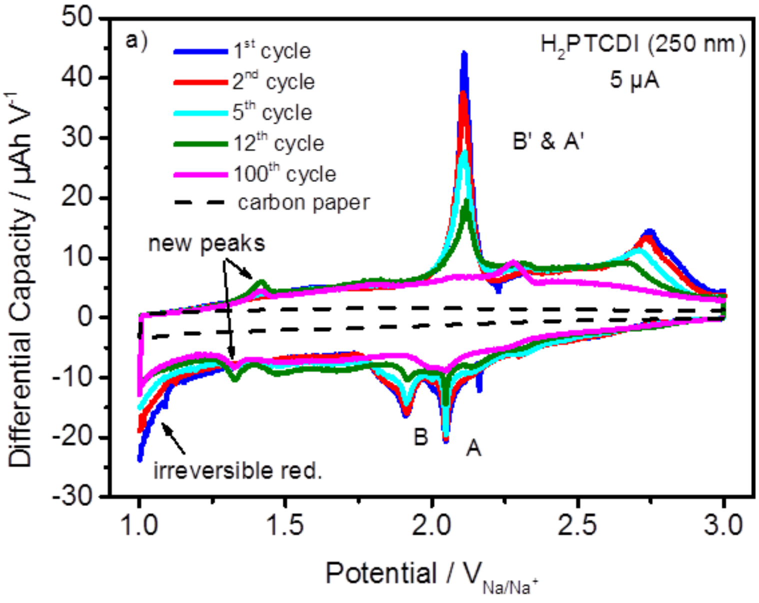

Figure S8. Differential capacity plot for the GCPL data obtained with an applied constant current of 5 µA of an initial 250 nm H_2PTCDI composite electrode from its first to its 100th cycle. The observed peaks are denoted as A, B and B'&A'. New additional peaks are highlighted with arrows. The differential capacity plot of carbon paper is illustrated as dashed line.

Experimental:

Material synthesis: Carbon paper discs with a diameter of 17 mm were punched out of a carbon paper sheet (MGL370, thickness: 0.3 mm). Commercially available 3,4,9,10-Perylene-Tetracarboxylic Diimide (H_2PTCDI, TCI Chemicals, >95%) was purified by vacuum sublimation in a tube furnace at 380 °C for 5 h prior to evaporation. Evaporation of 250 nm thin films of the PTCDI compounds was done under vacuum (~1-2*10^{-6} mbar) using a custom-built organic evaporation system from Vaksis R&D and Engineering, allowing precise rate control (1.2 Å s^{-1}) and material heating (at 340 °C). By using the electrode diameter (17 mm) and the evaporated film thickness (250 nm) a total active electrode mass of 90.8 µg can be calculated. This contributes only about 0.21% to the overall electrode mass of 42.5 mg.

Electrode Characterization: Scanning electron microscopy (SEM) images were recorded on a JEOL JSM-6360 LV machine at an acceleration voltage of 7 kV under high vacuum conditions. Raman spectra were recorded on an Olympus BX40 micro-Raman instrument equipped with a He/Ne laser (632.8 nm).

Battery assembling and electrochemical measurements: The electrochemical measurements were carried out in a three electrode ECC-Ref Cell (El-Cell) using a Biologic VMP3 potentiostat at room temperature. Sodium metal (Na rod in paraffin oil, VWR, 99.5%) was used as counter and reference electrode and a glass fiber disc (Ø=18 mm, thickness 1.55 mm, El-Cell) as separator. The electrolyte (Solvonic, 99%) used was 1 M NaFSI (sodium bis(fluorosulfonyl)imide) in a 1:1 (v/v) mixture of ethylene carbonate (EC) and dimethylcarbonate (DMC). The cells were produced in an Ar-filled glove box (UNI-lab, MBraun) with a water and oxygen content below 0.1 ppm. CV measurements were performed in the potential window from 1.0 to 3.0 V vs. Na/Na^+. Galvanostatic cycling with potential limitation (GCPL) was carried out between 3.0 and 1.0 V vs. Na/Na^+ at different applied constant currents (5, 35, 70 and 210 µA). EIS was carried out in a frequency range from 100 kHz to 10 mHz.

Ultraviolet and visible spectroscopy (UV-vis) are recorded in 1 cm quartz glass cuvettes at 298 K by using a Cary 3G UV-vis spectro-photometer. The experiments were taken ex situ, in order to obtain the solubility of the active electrode material H_2PTCDI in their electrolytes (1 M NaFSI in a 1:1 (v/v), EC:DMC mixture) after 115 repeated GCPL cycles. Spectra are recorded in the $-\Delta T/T$ mode, using a pure 1 M NaFSI in a 1:1 (v/v), EC:DMC mixture as the reference spectrum.
